# Impact of Cardiac Troponin Elevation on Mortality of Patients with Acute Heart Failure: Insights from the Korea Acute Heart Failure (KorAHF) Registry

**DOI:** 10.3390/jcm11102800

**Published:** 2022-05-16

**Authors:** Nuri Lee, Jae Yeong Cho, Kye Hun Kim, Hyung Yoon Kim, Hyun-Jai Cho, Hae-Young Lee, Eun-Seok Jeon, Jae-Joong Kim, Myeong-Chan Cho, Shung Chull Chae, Sang Hong Baek, Seok-Min Kang, Dong-Ju Choi, Byung-Su Yoo, Byung-Hee Oh, on behalf of the KorAHF Investigators

**Affiliations:** 1 Department of Cardiovascular Medicine, Chonnam National University Hwasun Hospital, Hwasun 58128, Korea; nurilee.md@gmail.com; 2 Department of Cardiovascular Medicine, Chonnam National University Medical School, Gwangju 61469, Korea; jaeycho@chonnam.edu; 3 Department of Cardiovascular Medicine, Chonnam National University Hospital, Gwangju 61469, Korea; medoc7@gmail.com; 4 Department of Internal Medicine, Seoul National University Hospital, Seoul 03080, Korea; hyunjaicho@snu.ac.kr (H.-J.C.); hylee612@snu.ac.kr (H.-Y.L.); ohbhmed@snu.ac.kr (B.-H.O.); 5 Department of Internal Medicine, Sungkyunkwan University College of Medicine, Seoul 16419, Korea; eunseok.jeon@samsung.com; 6 Department of Internal Medicine, Asan Medical Center, University of Ulsan College of Medicine, Seoul 05505, Korea; jjkim@amc.seoul.kr; 7 Department of Internal Medicine, Chungbuk National University College of Medicine, Cheongju 28644, Korea; mccho@chungbuk.ac.kr; 8 Department of Internal Medicine, Kyungpook National University College of Medicine, Daegu 37224, Korea; scchae@knu.ac.kr; 9 Department of Internal Medicine, College of Medicine, The Catholic University of Korea, Seoul 06591, Korea; whitesh@catholic.ac.kr; 10 Department of Internal Medicine, Yonsei University College of Medicine, Seoul 03722, Korea; smkang@yumc.yonsei.ac.kr; 11 Department of Internal Medicine, Seoul National University Bundang Hospital, Seongnam 13620, Korea; djchoi@snubh.org; 12 Department of Internal Medicine, Yonsei University Wonju College of Medicine, Wonju 26426, Korea; yubs@yonsei.ac.kr

**Keywords:** acute heart failure, etiology, cardiac troponin, prognosis

## Abstract

We aimed to conduct the largest study evaluating the impact of cardiac troponin (TnI) status on mid- and long-term mortality in patients admitted for acute heart failure (AHF) as compared between patients with ischemic (IHF) vs. non-ischemic heart failure (non-IHF). Among 5625 patients from the Korea Acute Heart Failure (KorAHF) registry, 4396 eligible patients with TnI measurement were analyzed. The patients were included on admission with the diagnosis of AHF, and TnI level was measured on the day of admission. A TnI value of <0.05 ng/mL was considered normal. The patients were divided into four groups according to the etiology of heart failure and the status of TnI: non-IHF with normal TnI (*n* = 1009) vs. non-IHF with elevated TnI (*n* = 1665) vs. IHF with normal TnI (*n* = 258) vs. IHF with elevated TnI (*n* = 1464). The primary outcome was death from all causes according to the etiology (non-IHF vs. IHF) and TnI elevation during the entire follow-up period of 784 days (IQR 446–1116). Elevation of TnI was observed in 71.2% of all patients with AHF. Patients with IHF had higher all-cause mortality compared to those with non-IHF. Elevated TnI was associated with higher 90-day and post-90-day mortality in the non-IHF group. IHF as compared to non-IHF and elevation of TnI were independent predictors of mortality also in the adjustment analysis. In the IHF group, however, elevated TnI had a higher mortality with only 90-day follow-up (18.6% vs. 25.9%, log-rank *p* < 0.001), not in the post-90-day follow-up (31.1% vs. 32.5%, log-rank *p* = 0.799). In conclusion, elevated TnI in patients with heart failure is associated with increased all-cause mortality regardless of the etiology of HF. Elevation of TnI was associated to a higher post-90 day mortality in patients with non-IHF but not in patients with IHF.

## 1. Introduction

Heart failure (HF) is a common clinical syndrome caused by various cardiac diseases. It is characterized by typical symptoms like breathlessness, leg swelling, and fatigue with signs such as jugular venous engorgement, pulmonary rales, and peripheral edema. The overall prevalence of HF has been increasing due to improvements in treatment and mortality for many cardiac diseases such as myocardial infarction, yet mortality remains significant [1,2,3,4,5,6,7]. The prevalence of HF has been increasing consistently in Korea from 0.77% in 2002 to 2.24% in 2018 with an aging society [8,9]. Between 2002 and 2018, the number of hospitalizations for HF increased 3.5-fold [10]. Thus, a continued increase in HF hospitalization and mortality is expected, and more precise risk stratification of the patients with HF is required.

Initial evaluation of HF includes identification of the etiology of the underlying cardiac dysfunction [11,12] as subsequent treatment might be determined by the cause of HF. HF from ischemic etiology could appear as a complication of undiagnosed or untreated CAD or may result as sequelae of a large myocardial infarction, even despite adequate treatment including percutaneous coronary intervention. On the other hand, HF from non-ischemic etiology include cardiomyopathies, hypertension, significant valvular diseases, tachyarrhythmias, infiltrative myocardial diseases, cardiotoxic chemotherapy-related HF, and infective disease such as human immunodeficiency virus (HIV) infection. The composition and the proportion of each cause of HF vary according to geography. It is known that CAD and hypertension are the prominent underlying cause of HF in Western and developed countries [13].

Cardiac troponin (cTn) is a regulatory protein controlling the calcium-mediated interaction between actin and myosin, which is used as a primary biomarker for the diagnosis of myocardial infarction [14]. But cTn can also be elevated in many other cardiac conditions, including acute and chronic heart failure [15,16]. The potential mechanisms of cTn elevation in HF are suggested as subendocardial ischemia, elevated filling pressure, and increased myocardial wall stress [17,18,19]. In patients with acute heart failure (AHF), cTn elevation has been reported to have a clinical correlation with increased mortality and readmission rates [14,15,20,21,22]. However, the comparison between the HF from ischemic cause and non-ischemic cause in regards to elevation of TnI has not been studied.

Several studies about Korean heart failure patients reported the proportion of the different etiologies of HF and outcomes of them but the association between cTN elevation and outcome is not reported [4,23,24,25]. Therefore, we aimed to investigate the prevalence and the impact of TnI elevation on mortality in Korean patients with ischemic and non-ischemic AHF. To the best of our knowledge, the present study is the largest study evaluating the impact of cTn status on mid- and long-term mortality in patients with AHF according to the etiology of HF.

## 2. Materials and Methods

### 2.1. Study Population

The Korea Acute Heart Failure (KorAHF) registry was designed to build a prospective, multi-center database to find clinical characteristics and outcomes of AHF in Korea. Patients hospitalized for AHF from 10 tertiary referral hospitals in Korea between March 2011 and February 2014 were consecutively enrolled. Patients who were more than 18 years old and had signs or symptoms of HF and one of the following criteria are eligible for the study: (i) lung congestion or (ii) objective findings of left ventricular (LV) systolic dysfunction or structural heart disease. Lung congestion was defined as ”congestion” on a chest X-ray or as rales on physical examination. Both a de novo (newly developed AHF in a patient without known cardiac dysfunction) and acute decompensated state of chronic heart failure (CHF) were included in the registry [26]. Structural heart disease was defined as any of the following: ischemic heart disease (IHF), congestive heart failure, valvular heart disease, cardiomyopathy, hypertension with left ventricular hypertrophy, hypertension with left ventricular dysfunction, left atrial enlargement, or conduction system disease. IHF was defined as the patients with prior MI and accompanied echocardiographic evidence of wall motion abnormality in infarcted myocardium or the patients without prior MI and documented severe obstructive coronary artery disease in coronary angiography, CT coronary angiography, or myocardial SPECT. These were evaluated by the physician taking care of the patient. The previous medical history was assessed by administering the questionnaire to the study subjects and also from available medical records. Written informed consent was obtained from each patient. If patients were unable to give consent due to disease severity, informed consent was obtained from a relative or legal representative. The study protocol of the KorAHF had been approved by the Institutional Review Board of each participating center. The detailed method of data recording is described elsewhere [26].

A total of 5625 patients were enrolled in the KorAHF registry. The patients were included on admission with the diagnosis of AHF, and TnI level was measured on the day of admission and at the discretion of the physician thereafter during the hospitalization. The highest value of TnI during the first two days of initial hospitalization was used in the analysis. Among them, an eligible 4396 patients were analyzed after the exclusion of 1229 patients whose initial TnI values were missed or extremely high at over 250 ng/mL. The TnI value of <0.05 ng/mL was considered normal and ≥0.05 ng/mL elevated. The patients were divided into four groups according to the etiology of heart failure (ischemic heart failure (IHF) vs. non-IHF) and the status of TnI (normal <0.05 ng/mL vs. elevated 0.05 ng/mL–250 ng/mL); non-IHF with normal TnI (*n* = 1009, median age 72 years, 446 males) vs. non-IHF with elevated TnI (*n* = 1665, median age 70 years, 812 males) vs. IHF with normal TnI (*n* = 258, median age 72 years, 258 males) vs. IHF with elevated TnI (*n* = 1464, median age 74 years, 895 males).

### 2.2. High-Sensitivity Troponin-I (TnI) Analysis

The TnI was measured and controlled during hospitalization at the discretion of the physician. The assay platform was chosen between the ADVIA Centaur system from Siemens and the ARCHITECT STAT system from Abbott by the laboratory setting of each attending center. Because the reference range of each diagnostic platform was different (as <0.047 ng/mL in Centaur and <0.026 ng/mL in Architect), a predefined cutoff value was set as 0.050 ng/mL or higher. The limit of quantification was similar between the two platforms, as 0.0025 ng/mL in Centaur and 0.0032 ng/mL in Architect.

In addition, the assay range of the ADVIA Centaur system from Siemens is 2.50–25,000 ng/L and the ARCHITECT STAT system from Abbott is 3.50–5000 ng/L. But actually, in practice, the ADVIA Centaur system can report the test results up to 250,000 ng/L (250 ng/mL). Thus, we had considered extremely high values above 250 ng/mL as outliers, such as unit error.

### 2.3. Outcome Measures

The primary outcome was death from all causes according to the etiology (non-IHF vs. IHF) and TnI elevation (<0.05 vs. 0.05–250 ng/mL). The secondary outcomes were both 90-day and post-90-day all-cause death according to TnI elevation in both IHF and non-IHF groups. The outcome data for subjects who had not been followed up have been ascertained by a telephone interview. In addition, the outcome data for patients lost to follow-up were collected from the National Health Insurance Service or the National Death Records.

### 2.4. Statistical Analysis

Categorical variables are given as numbers and percentages and compared by the Kruskal–Wallis test. Continuous variables are described as mean ± standard deviation in the case of normally distributed variables or median with interquartile range (IQR) for not normally distributed data. The analysis of variance (ANOVA) was used for the assessment of between-group differences for the normally distributed variables and the Kruskal–Wallis test was used for all other variables. Bonferroni’s correction was applied to the post hoc analysis of the between-group comparisons to allow six comparisons for each variable. A *p*-value equal to or less than 0.05 was considered statistically significant. All reported *p* values are two-sided. Cumulative event rates were estimated by using the Kaplan–Meier method and compared by the log-rank test. The Cox proportional hazards model was used for the identification of the factors associated with all-cause mortality. Factors associated with mortality with a *p*-value of less than 0.2 in the univariate analysis were entered in the multivariate model. Age, sex, previous history of admission due to HF, hypertension, diabetes mellitus, chronic kidney disease (CKD), chronic obstructive pulmonary disease (COPD), cerebrovascular accident (CVA), prior history of coronary artery disease (CAD) or myocardial infarction (MI), malignancy, initial systolic blood pressure (SBP), initial left ventricular ejection fraction (LVEF), initial serum sodium (Na), initial serum creatinine (Cr), atrial fibrillation (AF) at admission, medication at discharge, including angiotensin-converting enzyme inhibitor (ACEI)/angiotensin II receptor blocker (ARB), beta-blockers (BB), or aldosterone antagonist (AA) were included as covariates in multivariate COX proportional-hazard model. Irrelevant factors were removed by means of a backward-selection procedure. Landmark analysis was performed at 30 days by the etiology of the HF and the status of TnI. Additional analysis was performed comparing all-cause death according to etiology of the HF and the elevation of TnI at both 90-days and post-90-day follow-up. Analyses were performed with R Statistical Software version 4.0.1 (R Foundation for Statistical Computing, Vienna, Austria).

## 3. Results

The number of patients without measurement of TnI was 1170. Among the 4455 patients who had the result of TnI, we excluded 59 patients because they had a TnI value above 250 ng/mL. Finally, 4396 patients were included in the analyses. The median follow-up duration was 784 (IQR 446–1116) days. The median age of the patients was 72 (IQR 61–79) years, 52.6% were male, and 71.2% showed elevated TnI (62.3% in IHF vs. 85.0% in non-IHF).

### 3.1. Baseline Characteristics of the Patients

The four groups had significant differences in baseline characteristics as seen in Table 1. The patients with IHF were older and more likely to be male, had hypertension, diabetes, chronic kidney disease (CKD), a history of CVA, prior coronary artery disease (CAD) or myocardial infarction (MI), high initial SBP, a high level of TnI upon admission, a low initial LVEF, and a high level of initial serum Cr compared to those with non-IHF (Table 1). The etiology of HF was ischemia for 1722 patients (39.2%) whereas in patients with non-IHF, the specific etiologies included cardiomyopathies (31.9%), valvular heart diseases (21.6%), and tachycardia-associated cardiomyopathy (18.4%). The proportion of poor prognosis groups such as infiltrative heart disease (2.3%) was very small compared with idiopathic cardiomyopathies (Figure S2).

### 3.2. All-Cause Mortality According to the Etiology of AHF and the Status of TnI

**Figure 1:**
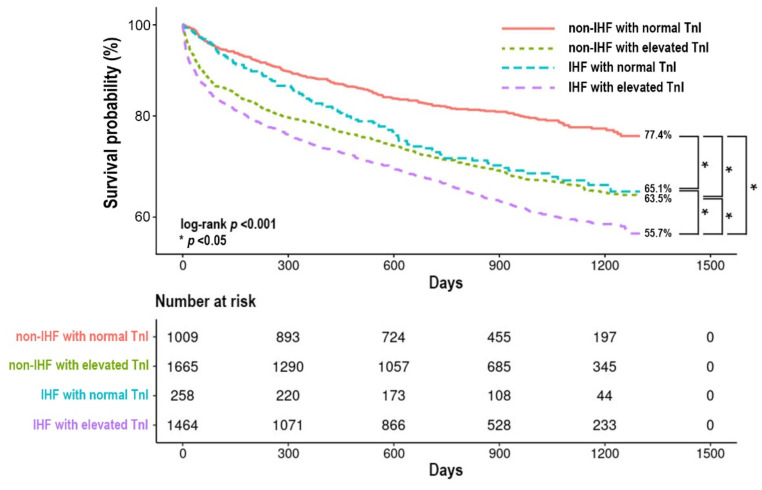
Kaplan–Meier survival curve according to the etiology of HF and status of TnI. IHF, ischemic heart failure; TnI, high-sensitivity troponin-I.

Patients in the IHF group showed more frequent TnI elevation (0.05–250 ng/mL) than those in the non-IHF group (85.0% vs. 62.3%, *p* < 0.001). All-cause mortality of a total of 4396 patients was 35.8% during a median follow-up of 784 days (IQR 446–1116 days). The four groups showed significantly different overall mortality. Kaplan–Meier survival curves showed the highest overall mortality in the IHF with elevated TnI group followed by the two groups—non-IHF with elevated TnI group and IHF with normal TnI group—that had similar mortality, whereas patients with non-IHF with normal TnI had the lowest mortality (Figure 1). Multivariate analysis using the Cox proportional-hazards model showed that grouping by the etiology of HF and the status of TnI was an independent predictor for all-cause mortality during the entire follow-up (Table 2). Compared to non-IHF with normal TnI as a reference, the adjusted hazard ratio (HR) for all-cause mortality of the non-IHF with elevated TnI group was 1.60 (95% CI 1.36–1.88, *p* < 0.001), 1.44 for the IHF with the normal TnI group (95% CI 1.12–1.85, *p* = 0.005), and 1.88 for the IHF with elevated TnI group (95% CI 1.60–2.21, *p* < 0.001), respectively. The factors associated with increased all-cause mortality were the etiology of HF and TnI elevation, age ≥ 65 years, previous history of admission due to HF, CKD, COPD, history of CVA, malignancy, initial SBP < 110 mmHg, initial LVEF < 40%, initial serum Na < 135 mEq/L, and initial serum Cr ≥ 2.0 mg/dL. The use of ACEI/ARB and BB at discharge were associated with better overall survival (Figure S1).

### 3.3. The 90-Day and Post-90-Day Mortality According to the Status of TnI in Non-IHF

**Figure 2:**
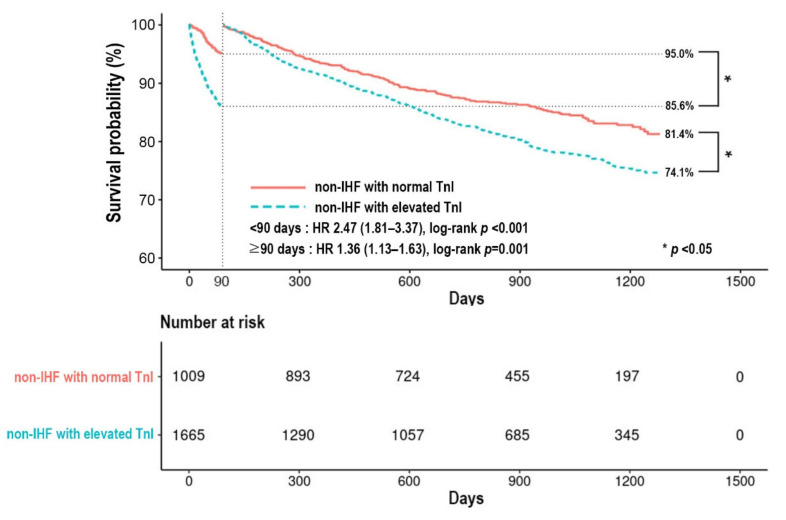
Kaplan–Meier survival curve according to status of TnI in patients with non-IHF. IHF, ischemic heart failure; TnI, high-sensitivity troponin-I; HR, hazard ratio.

All-cause mortality of the 2674 patients with non-IHF was 31.3% during a median follow-up of 807 days (IQR 497–1142 days). The 90-day all-cause mortality was 5.0% in the non-IHF with normal TnI group and 14.4% in the non-IHF with elevated TnI group, respectively (Figure 2). In covariate-adjusted Cox proportional-hazard regression models, elevated TnI was associated with higher 90-day mortality (HR 2.48, 95% CI 1.82–3.37, *p* < 0.001) (Table S2). The post-90-day all-cause mortality was 18.6% in the non-IHF with normal TnI group and 25.9% in the non-IHF and elevated TnI group. Elevation of TnI was associated with higher mortality in Cox proportional-hazard regression models with HR of 1.38 (95% CI 1.14–1.67, *p* < 0.001). In covariate-adjusted Cox proportional-hazard regression models, it was revealed that factors associated with higher 90-days mortality were elevated TnI, age ≥ 65 years, prior MI, CKD, malignancy, initial SBP < 110 mmHg, initial serum Na < 135 mEq/L and use of ACEI/ARB, BB, AA at discharge were associated with lower 90-day mortality (Table S2). An increased post-90-day all-cause mortality was associated with elevated TnI, age ≥ 65 years, previous history of admission due to HF, CKD, and malignancy, whereas the use of BB at discharge was associated with a lower post-90-day mortality (Table S2).

### 3.4. The 90-Day and Post-90-Day Mortality According to the Status of TnI in IHF

**Figure 3:**
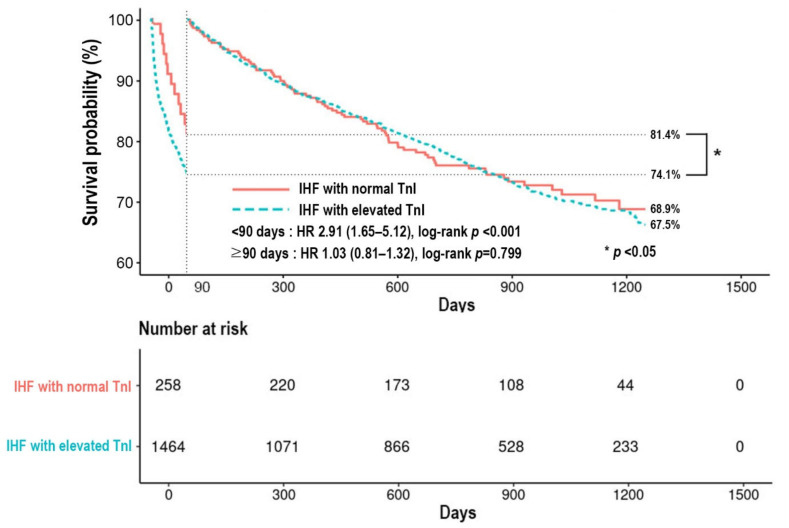
Kaplan–Meier survival curve according to status of TnI in patients with IHF. IHF, ischemic heart failure; TnI, high-sensitivity troponin-I; HR, hazard ratio.

The median follow-up duration was 756 days (IQR 299–1068 days) in patients with ischemic heart failure. The overall all-cause mortality of the 1722 patients with IHF was 42.9%. The 90-day all-cause mortality was 18.6% in IHF with normal TnI group and 25.9% in the IHF with elevated TnI group (Figure 3). This difference was significant in covariate-adjusted Cox proportional-hazard regression models with an HR of 2.91 (95% CI 1.65–5.12, *p* < 0.001) (Table S2). However, the post-90-day all-cause mortality was 31.1% and 32.5% in the IHF with normal TnI group and the IHF with elevated TnI group, respectively. Univariate analysis showed no statistical difference between the two groups with an HR of 1.03 (95% CI 0.81–1.32, *p* = 0.799). Elevated 90-day mortality was associated with elevated TnI, age ≥ 65 years, CKD, COPD, initial SBP < 110 mmHg, initial LVEF < 40%, initial serum Na < 135 mEq/L and initial serum Cr ≥ 2.0 mg/dL in covariate-adjusted Cox proportional-hazard regression model. The use of ACEI/ARB and BB was associated with lower overall mortality. Increased post-90-day mortality was associated with factors like age ≥ 65 years, the previous history of admission due to HF, CKD, COPD, history of CVA, malignancy, initial LVEF < 40% and initial serum Na < 135 mEq/L (Table S2).

### 3.5. The Relationship between the Degree of TnI Elevation and Mortality

The total number of study patients were divided in quartiles (Q) according to TnI level (Q1 < 0.05 vs. Q2 0.05–0.50 vs. Q3 0.50–5.00 vs. Q4 > 5.00 ng/mL). The number of patients were 258 vs. 559 vs. 388 vs. 517 in IHF and 1099 vs. 1088 vs. 332 vs. 245 in non-IHF, respectively, as shown in Figures S3 and S4 (Q1 vs. Q2 vs. Q3 vs. Q4). Q1 showed the most favorable outcome in patients with IHF and non-IHF. In patients with non-IHF, however, Q4 also displayed a survival curve similar to Q1 (Figure S4). This might be attributed to the fact that patients with myocarditis, a reversible cause of AHF were more frequently allocated to Q4 than those with cardiomyopathy (52.9% vs. 6.3%, *p* < 0.001).

## 4. Discussion

We investigated the prevalence of TnI elevation and the impact of elevated TnI on mortality in Korean patients with ischemic and non-ischemic AHF. In the KorAHF registry of 5625 patients, TnI was measured in 4396 patients (78.2%) as part of routine care and 39.2% and 60.8% of them had IHF and non-IHF respectively. In addition, 71.2% of the total patients were found to have elevated TnI values whereas only 29.2% of the study patients had a history of CAD or MI. In this analysis of AHF patients categorized according to the etiology of HF and status of cTn, we identified different prognoses among the groups. All-cause mortality in the IHF with elevated TnI group was higher than any other group whereas non-IHF with normal TnI group showed the lowest all-cause mortality. In addition, the 90-day mortality was higher in patients with elevated TnI than in those with normal TnI for both patients with non-IHF and IHF. However, the post-90-day mortality was higher in patients with elevated TnI in the non-IHF group only. The mortality of patients with non-IHF with elevated TnI was similar to that of patients with IHF with normal TnI. To the best of our knowledge, the present study is the largest study evaluating the impact of cTn status on mid- and long-term mortality in patients with AHF according to the etiology of HF. Simple evaluation of admission TnI may be useful in identifying the high-risk groups of AHF or in the risk stratification of AHF, especially in non-IHF.

After the identification of cTn, their use has increased markedly for the detection of acute myocardial infarction due to high specificity for ongoing myocardial damage [27,28]. But cTn may also be elevated in other cardiac conditions such as acute and chronic heart failure [20]. Although the precise mechanism and the significance of an elevated TnI in acute and chronic heart failure are not clear, release of cTn might be caused by both acute and chronic myocardial stress, as well as chronic subclinical subendocardial ischemia or directly related to injury of cardiomyocytes [29]. In the highly-sensitive cTn era, detectable cTn using these highly sensitive cTn assays in non-HF patients is quite common, and elevated values are associated with increased all-cause death, cardiovascular death, and incident HF after adjusting renal function, N-terminal pro-B-type natriuretic peptide (NT-proBNP) and high-sensitivity C-reactive protein (CRP) [30,31,32].

In the present study, patients with HF due to non-ischemic causes comprised 60.8% of the total study population. In a study of 1230 patients with cardiomyopathy with various etiologies, Felker et al. [33] showed that the underlying causes of heart failure had prognostic value. Peripartum cardiomyopathy and cardiomyopathy due to hypertension showed 69% and 26% decreased HR for death compared to idiopathic cardiomyopathy whereas cardiomyopathy due to ischemic heart disease showed 52% increased HR for death. These results are consistent with the findings of the present study in terms of the finding that the overall mortality of the non-IHF group was better than the IHF group. However, in the deeper analysis according to the status of TnI, there emerge meaningful differences among the groups. During the entire follow-up period, IHF had poorer outcomes than non-IHF, and the patients with elevated TnI were shown to have increased all-cause mortality compared to the patients with normal TnI. Furthermore, the serum level of TnI had a different impact on the outcomes at mid- and long-term follow-up between the non-IHF vs. IHF group. In the non-IHF group, the patients with elevated TnI had increased all-cause mortality at both 90-day and post-90-day follow-up. However, in the IHF group, only 90-day mortality was affected. One possible explanation would be that a significant number of patients with acute myocardial infarction and acute decompensated HF could have been included in the IHF with elevated TnI group. Many of these patients are indicated for revascularization treatment such as percutaneous coronary intervention (PCI) and showed high early mortality compared to non-candidates for PCI. But the long-term outcome might be not so poor in this group after successful PCI if their medical adherence was good. There are several studies investigating the clinical implications of cTn in HF. The ADHERE trial enrolled 61,397 patients who had conventional cTn level measurement on admission. Unlike the present study, only 6.2% of the patients had a positive value of cTn. However, they had higher rates of adverse events and mortality during hospitalization compared to the negative troponin group [19]. In the ASCEND-HF study, sensitive cTn level testing (VITROS-TOP) was used as a cTn assay and positive troponin levels were seen in 50% of the total of 808 patients with AHF. Patients with elevated cTn more often had HF worsening during hospitalization but elevation of cTn was not an independent predictor of adverse outcomes at 90 days and 1 year [34]. Arenja N et al. analyzed 667 patients who visited an emergency department for acute dyspnea and 377 (57%) patients were diagnosed with AHF retrospectively. The authors report that high-sensitivity cTn levels correlated with 30-day and 1-year mortality [35]. Nevertheless, none of the above studies demonstrated the different impact of elevated cTn according to ischemic etiology in AHF as indicated by our study. In addition, the strength of our data comes from a large-scale cohort registry of AHF, which did not have any specific exclusion criteria. Our registry is the largest prospective cohort registry of AHF that used high-sensitivity cTn assays in routine practice. Furthermore, the data about mortality was reinforced by government-run insurance data.

This study has several limitations. First of all, this study used data from an observational registry. Many patients in the registry were excluded from the present study as an initial TnI was not available. Although we tried to adjust the influence of variables known to affect mortality such as age, hypertension, previous history of admission due to HF, COPD, initial low SBP, initial low LVEF, initial low serum Na and use of ACEI/ARB or BB at discharge, there are intrinsic limitations of observational data. Because the KorAHF registry has halted further follow-up at the moment, data beyond 4 years of follow-up was not available. Patients with normal EF could be included in non-IHF with the normal TnI group. This might be a potential confounding factor, though this category of patients does not comprise a major portion of the study population. Assay platforms used to measure high-sensitivity cTn were different among the participating centers. Hence, the higher cut-off value of the two assays was inevitably used. The HF rehospitalization rate according to the status of TnI was not included in the present analysis because it was difficult to collect admission data when patients were admitted to hospitals that did not participate in the KorAHF registry. Though the clinical significances of cTn in each study are slightly different, these differences might be due to different sample sizes, racial characteristics, and different composition of the etiology of HF. The number of patients with IHF and normal TnI was rather small, which needs caution in the interpretation of the results.

## 5. Conclusions

Patients with IHF had higher all-cause mortality compared to those with non-IHF. Elevated TnI of 0.05–250 ng/mL was associated with increased 90-day and post-90-day all-cause mortality in non-IHF. An elevated TnI level was associated with increased 90-day all-cause mortality, but not with post-90-day all-cause mortality in patients with IHF. These findings may be important for the prognostication of patients with AHF.

## Figures and Tables

**Table 1:** Baseline characteristics of patients in the four groups.

Characteristic	Non-IHF with Normal TnI (*n* = 1009)	Non-IHF with Elevated TnI (*n* = 1665)	IHF with Normal TnI (*n* = 258)	IHF with Elevated TnI (*n* = 1464)	*p*
Median age—yr	72 (59–78)	70 (56–78)	72 (66–79) ^†,§^	74 (66–80) ^‡,^*	<0.001
Male sex—no. (%)	446 (44.2)	812 (48.8)	159 (61.6) ^†,§^	895 (61.1) ^‡,^*	<0.001
Previous admission due to HF	279 (27.7)	529 (31.8)	109 (42.2) ^†,§^	418 (28.6) ^♭^	<0.001
Medical conditions—no. (%)					
Hypertension	550 (54.5)	863 (51.8)	178 (69.0) ^†,§^	1043 (71.2) ^‡,^*	<0.001
Diabetes	253 (25.1)	421 (25.3)	133 (51.6) ^†,§^	755 (51.6) ^‡,^*	<0.001
Chronic kidney disease	72 (7.1)	209 (12.6) ^¶^	35 (13.6) ^†^	285 (19.5) ^‡,^*	<0.001
Chronic obstructive pulmonary disease	124 (12.3)	200 (12.0)	28 (10.9)	146 (10.0)	0.216
Cerebrovascular accident	125 (12.4)	224 (13.5)	47 (18.2)	272 (18.6) ^‡,^*	<0.001
Prior coronary artery disease	101 (10.0)	149 (8.9)	196 (76.0) ^†,§^	837 (57.2) ^‡,^*^,^^♭^	<0.001
Prior myocardial infarction	36 (3.6)	55 (3.3)	141 (54.7) ^†,§^	513 (35.1) ^‡,^*^,^^♭^	<0.001
Heart failure	398 (39.5)	731 (43.9)	141 (54.7) ^†,§^	556 (38.0) *^,^^♭^	<0.001
Malignancy	89 (8.8)	151 (9.1)	18 (7.0)	100 (6.8)	0.095
Initial clinical findings					
Median SBP—mmHg	130 (112–150)	129 (109–151)	132 (113–153)	132 (113–153) *	0.004
Median LVEF—%	43.0 (28.0–57.1)	36.0 (24.6–52.0) ^¶^	36.0 (26.4–49.2) ^†^	34.4 (26.0–44.0) ^‡,^*	<0.001
Median TnI—ng/mL	0.02 (0.01–0.04)	0.10 (0.05–0.25) ^¶^	0.02 (0.01–0.04) ^§^	0.20 (0.20–1.74) ^‡,^*^,^^♭^	<0.001
Median serum Na—mEq/L	139 (136–141)	138 (135–140) ^¶^	138 (136–141) ^§^	138 (135–140) ^‡^	<0.001
Median serum Cr—mg/dL	0.94 (0.75–1.23)	1.08 (0.81–1.52) ^¶^	1.10 (0.86–1.44) ^†^	1.21 (0.90–1.80) ^‡,^*^,^^♭^	<0.001
Atrial fibrillation at admission	533 (52.8)	628 (37.7) ^¶^	92 (35.7) ^†^	231 (15.8) ^‡,^*^,^^♭^	<0.001
Procedures during the index hospitalization—no. (%)					
Coronary revascularization	1 (0.1)	25 (1.5)	28 (10.9) ^†§^	570 (39.0) ^‡,^*^,^^♭^	<0.001
Medication at discharge—no. (%)					
ACEI/ARB	692 (68.6)	1016 (61.0) ^¶^	196 (76.0) ^§^	964 (65.8) *^,^^♭^	<0.001
Beta-blocker	567 (56.2)	743 (44.6) ^¶^	159 (61.6) ^§^	804 (54.9) *	<0.001
Aldosterone antagonist	513 (50.8)	703 (42.2) ^¶^	122 (47.3)	523 (35.7) ^‡,^*^,^^♭^	<0.001

The numbers for continuous variables are median (IQR). ^¶^ *p* < 0.05 for non-IHF with normal TnI vs. non-IHF with elevated TnI. ^†^ *p* < 0.05 for non-IHF with normal TnI vs. IHF with normal TnI. ^‡^ *p* < 0.05 for non-IHF with normal TnI vs. IHF with elevated TnI. ^§^ *p* < 0.05 for non-IHF with elevated TnI vs. IHF with normal TnI. * *p* < 0.05 for non-IHF with elevated TnI vs. IHF with elevated TnI. ^♭^ *p* < 0.05 for IHF with normal TnI vs. IHF with elevated TnI. IHF, ischemic heart failure; TnI, high-sensitivity troponin-I; HF, heart failure; SBP, systolic blood pressure; LVEF, left ventricular ejection fraction; Na, sodium; Cr, creatinine; ACEI, angiotensin converting enzyme inhibitor; ARB, angiotensin II receptor blocker; IQR, interquartile range.

**Table 2:** Multivariate Cox proportional-hazards model for the factors associated with overall all-cause mortality.

Factor	Crude Hazard Ratio on Univariate Analysis (95% CI)	*p*	Adjusted Hazard Ratio on Multivariate Analysis (95% CI)	*p*
Groups		<0.001		<0.001
Non-IHF with normal TnI	1.00		1.00	
Non-IHF with elevated TnI	1.77 (1.52–2.07)	<0.001	1.60 (1.36–1.88)	<0.001
IHF with normal TnI	1.61 (1.26–2.06)	<0.001	1.44 (1.12–1.85)	0.005
IHF with elevated TnI	2.29 (1.97–2.66)	<0.001	1.88 (1.60–2.21)	<0.001
Demographics				
Age ≥ 65 year	2.59 (2.27–2.96)	<0.001	2.61 (2.25–3.02)	<0.001
Male sex	1.01 (0.91–1.11)	0.892		
Comorbidities				
Hypertension	1.38 (1.24–1.53)	<.0.001	1.10 (0.97–1.23)	0.128
Diabetes	1.32 (1.19–1.46)	<0.001		
Previous admission due to HF	1.74 (1.58–1.93)	<0.001	1.34 (1.20–1.49)	<0.001
Prior coronary artery disease	1.53 (1.38–1.69)	<0.001		
Prior myocardial infarction	1.52 (1.35–1.71)	<0.001		
Chronic kidney disease	2.01 (1.78–2.26)	<0.001	1.32 (1.12–1.55)	0.001
Chronic obstructive pulmonary disease	1.46 (1.27–1.68)	<0.001	1.27 (1.09–1.47)	0.002
Cerebrovascular accident	1.54 (1.36–1.74)	<0.001	1.23 (1.08–1.40)	0.002
Malignancy	1.67 (1.43–1.95)	<0.001	1.51 (1.28–1.77)	<0.001
Findings at admission				
SBP < 110 mmHg	1.31 (1.17–1.47)	<0.001	1.27 (1.12–1.44)	<0.001
LVEF < 40%	1.12 (1.01–1.24)	0.033	1.26 (1.13–1.41)	<0.001
Serum Na < 135 mEq/L	1.80 (1.61–2.01)	<0.001	1.56 (1.39–1.75)	<0.001
Serum Cr ≥ 2.0 mg/dL	1.95 (1.73–2.20)	<0.001	1.28 (1.09–1.50)	0.003
Atrial fibrillation	0.93 (0.83–1.03)	0.160		
Medication at discharge				
ACEI/ARB	0.56 (0.51–0.62)	<0.001	0.65 (0.58–0.72)	<0.001
Beta-blocker	0.60 (0.55–0.67)	<0.001	0.69 (0.62–0.77)	<0.001
Aldosterone antagonist	0.82 (0.74–0.91)	<0.001		

CI, confidence interval; IHF, ischemic heart failure; TnI, high-sensitivity troponin-I; HF, heart failure; SBP, systolic blood pressure; LVEF, left ventricular ejection fraction; Na, sodium; Cr, creatinine; ACEI, angiotensin converting enzyme inhibitor; ARB, angiotensin II receptor blocker.

## Data Availability

Data is contained within the article.

## Supplementary Materials

The following supporting information can be downloaded at https://www.mdpi.com/article/10.3390/jcm11102800/s1, Figure S1: Multivariate Cox proportional hazards model for the factors associated with overall all-cause mortality. Figure S2: Composition of the etiologies of acute heart failure in KorAHF registry. Table S1: Crude hazard ratio for the factors associated with 90-day and post-90-day all-cause mortality on univariate analysis. Figure S3: Kaplan-Meier survival curve according to the degree of TnI elevation in patients with ischemic heart failure. TnI, high-sensitivity troponin-I. Figure S4: Kaplan-Meier survival curve according to the degree of TnI elevation in patients with non-ischemic heart failure. TnI, high-sensitivity troponin-I. Table S2: Multivariate Cox proportional-hazards model for the factors associated with 90-day and post-90-day all-cause mortality.
