# Supplementary material for: Impact of Cardiac Troponin Elevation on Mortality of Patients with Acute Heart Failure: Insights from the Korea Acute Heart Failure (KorAHF) Registry
Source: J Clin Med. 2022 May 16;11(10):2800. doi: 10.3390/jcm11102800 (PMC9145996; doi:10.3390/jcm11102800)
Supplement: Supplementary file 1 [file jcm-11-02800-s001.zip › jcm-1695411-supplementary.pdf]

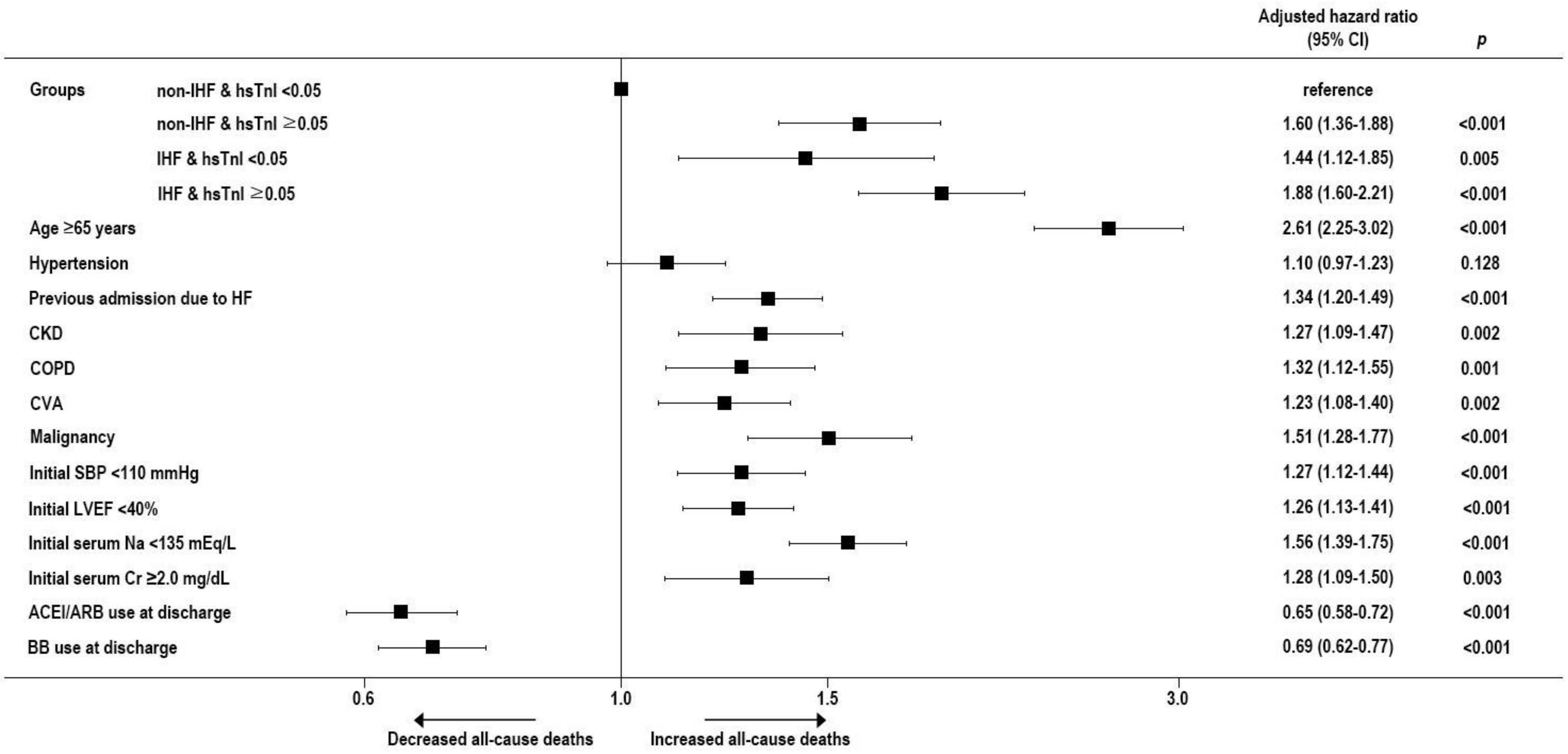

Figure S1. Multivariate Cox proportional hazards model for the factors associated with all-cause mortality. IHF, ischemic heart failure; TnI, high-sensitivity troponin-I; HF, heart failure; CKD, chronic kidney disease; COPD, chronic obstructive pulmonary disease; CVA, cerebrovascular accident; SBP, systolic blood pressure; LVEF, left ventricular ejection fraction; ACEI, angiotensin-converting enzyme inhibitor; ARB, angiotensin receptor blocker; BB, beta-blocker

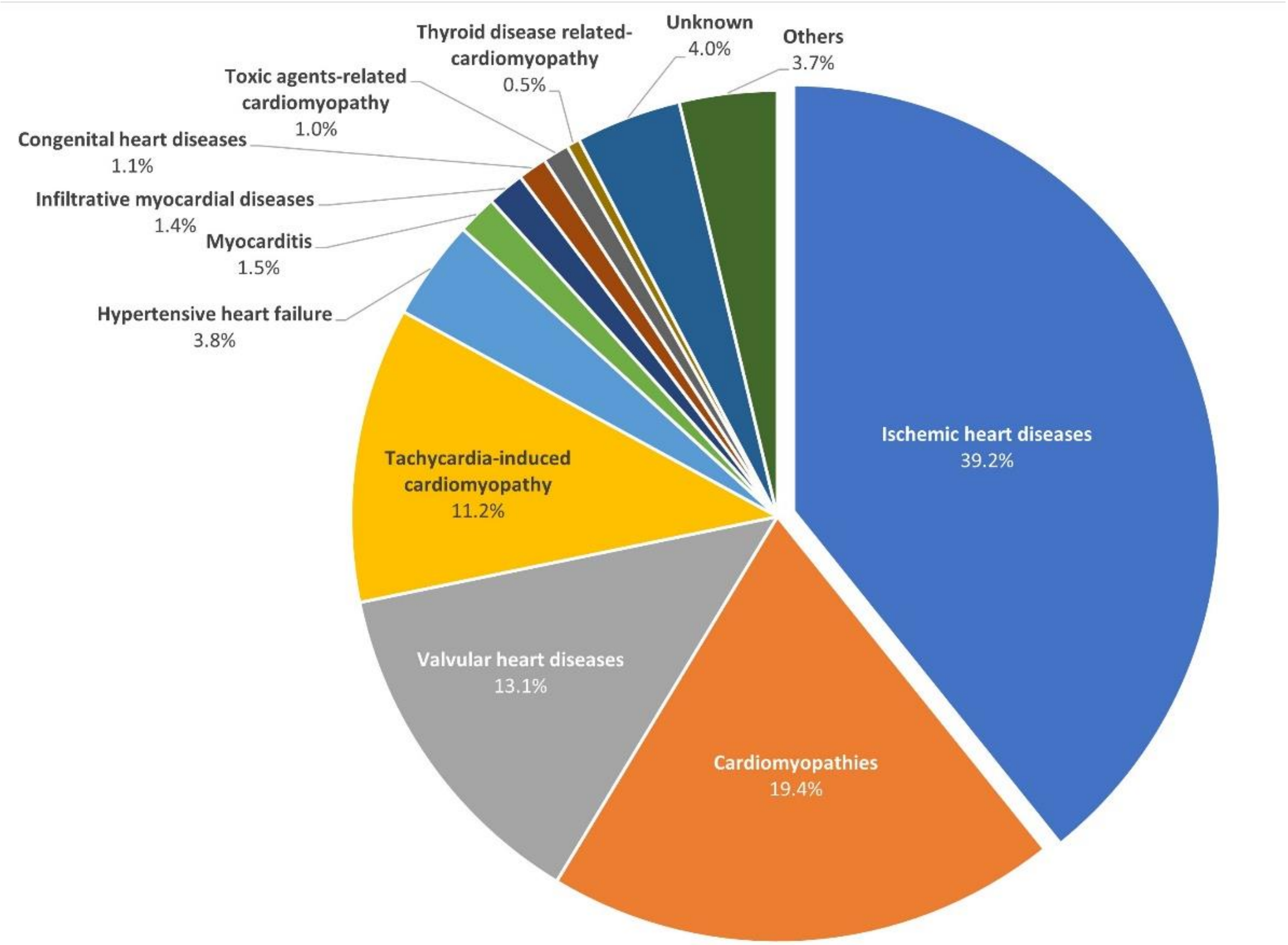

Figure S2. Composition of the etiologies of acute heart failure in KorAHF registry

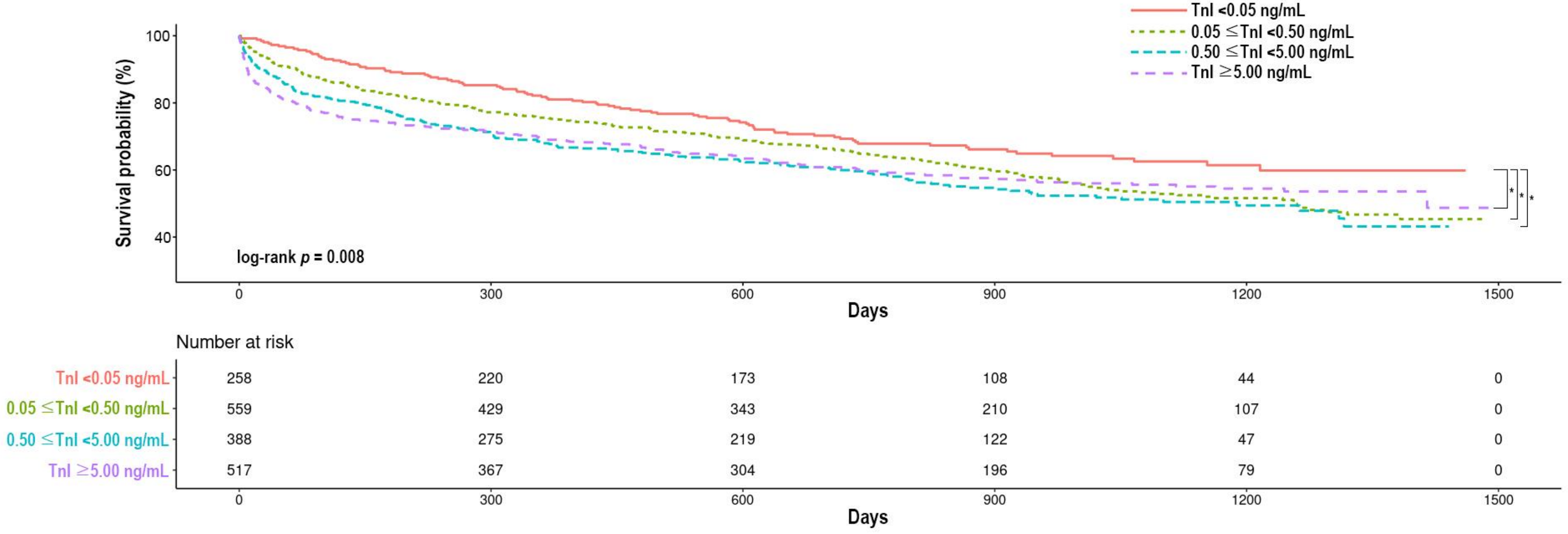

Figure S3. Kaplan-Meier survival curve according to the degree of TnI elevation in patients with ischemic heart failure. TnI, high-sensitivity troponin-I. * $p < 0.05$.

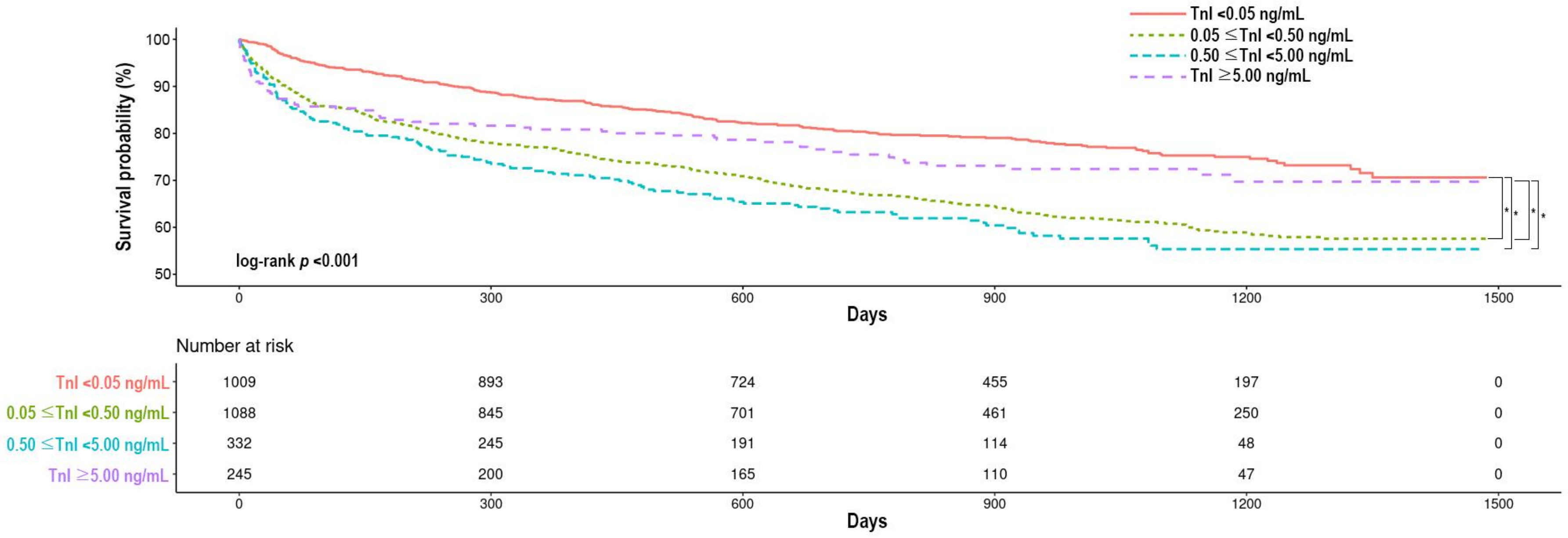

Figure S4. Kaplan-Meier survival curve according to the degree of TnI elevation in patients with non-ischemic heart failure. TnI, high-sensitivity troponin-I. * $p < 0.05$.

Table S1. The unadjusted hazard ratio for the factors associated with 90-day and post-90-day all-cause mortality on univariate analysis

	**Non-ischemic heart failure**				**Ischemic heart failure**			
	**90-day All-cause Death**		**Post-90-day All-cause Death**		**90-day All-cause Death**		**Post-90-day All-cause Death**	
	**Crude HR (95% CI)**	***p***	**Crude HR (95% CI)**	***p***	**Crude HR (95% CI)**	***p***	**Crude HR (95% CI)**	***p***
**Groups**								
Non-IHF with normal hsTnI	1.00		1.00					
Non-IHF with elevated hsTnI	3.08 (2.27-4.17)	<0.001	1.41 (1.18-1.68)	<0.001				
IHF with normal hsTnI					1.00		1.00	
IHF with elevated hsTnI					3.47 (2.02-5.93)	<0.001	1.03 (0.81-1.32)	0.799
**Demographics**								
Age ≥65 years	1.89 (1.44-2.47)	<0.001	3.04 (2.45-3.77)	<0.001	2.26 (1.56-3.27)	<0.001	2.39 (1.83-3.12)	<0.001
Male sex	1.10 (0.87-1.38)	0.442	0.79 (0.67-0.94)	0.007	0.95 (0.74-1.21)	0.651	1.10 (0.91-1.33)	0.326
**Comorbidities**								
Hypertension	1.08 (0.85-1.36)	0.542	1.41 (1.19-1.68)	<0.001	1.00 (0.77-1.30)	0.988	1.54 (1.24-1.91)	<0.001
Diabetes	1.18 (0.91-1.53)	0.203	1.39 (1.16-1.67)	<0.001	0.88 (0.69-1.11)	0.272	1.25 (1.04-1.50)	0.016
Previous admission due to HF	1.81 (1.44-2.29)	<0.001	2.15 (1.81-2.54)	<0.001	1.09 (0.84-1.40)	0.529	1.68 (1.40-2.02)	<0.001
Prior CAD	1.52 (1.08-2.14)	0.016	1.48 (1.14-1.91)	0.003	0.92 (0.72-1.18)	0.515	1.47 (1.21-1.78)	<0.001
Prior MI	2.12 (1.33-3.37)	0.002	1.52 (1.00-2.31)	0.050	0.95 (0.74-1.22)	0.705	1.35 (1.12-1.62)	0.001
CKD	2.01 (1.50-2.71)	<0.001	2.28 (1.83-2.85)	<0.001	1.37 (1.03-1.81)	0.031	1.92 (1.56-2.36)	<0.001
COPD	1.44 (1.05-1.96)	0.023	1.47 (1.17-1.85)	0.001	1.88 (1.36-2.60)	<0.001	1.40 (1.05-1.86)	0.021
CVA	1.49 (1.10-2.01)	0.010	1.52 (1.22-1.91)	<0.001	1.35 (1.01-1.79)	0.041	1.53 (1.24-1.90)	<0.001
Malignancy	1.90 (1.38-2.62)	<0.001	1.63 (1.26-2.11)	<0.001	1.78 (1.22-2.61)	0.003	1.67 (1.22-2.28)	0.001
**Findings at admission**								
SBP <110 mmHg	2.54 (2.01-3.21)	<0.001	0.95 (0.77-1.16)	0.593	2.02 (1.56-2.62)	<0.001	0.91 (0.71-1.17)	0.474
LVEF <40%	1.07 (0.84-1.37)	0.568	0.74 (0.62-0.88)	<0.001	1.72 (1.29-2.30)	<0.001	1.37 (1.13-1.67)	0.002
Serum Na <135 mEq/L	2.36 (1.86-2.99)	<0.001	1.78 (1.48-2.15)	<0.001	1.67 (1.28-2.16)	<0.001	1.58 (1.28-1.94)	<0.001
Serum Cr ≥2.0 mg/dL	1.85 (1.37-2.48)	<0.001	1.97 (1.58-2.45)	<0.001	1.94 (1.50-2.51)	<0.001	1.71 (1.39-2.11)	<0.001

Atrial fibrillation	0.88 (0.70-1.12)	0.294	1.08 (0.91-1.27)	0.396	1.24 (0.93-1.66)	0.144	1.01 (0.80-1.28)	0.913
**Medication at discharge**								
ACEI/ARB	0.29 (0.23-0.37)	<0.001	0.78 (0.66-0.93)	0.006	0.26 (0.20-0.33)	<0.001	0.87 (0.72-1.06)	0.178
BB	0.36 (0.28-0.47)	<0.001	0.73 (0.62-0.87)	<0.001	0.29 (0.22-0.38)	<0.001	0.83 (0.69-1.00)	0.050
AA	0.61 (0.48-0.78)	<0.001	0.97 (0.82-1.14)	0.684	0.58 (0.44-0.76)	<0.001	1.06 (0.88-1.27)	0.573

HR, hazard ratio; IHF, ischemic heart failure; hsTnI, high-sensitivity troponin-I; HF, heart failure; CKD, chronic kidney disease; COPD, chronic pulmonary obstructive disease; CVA, cerebrovascular accident; CAD, coronary artery disease; MI, myocardial infarction; SBP, systolic blood pressure; LVEF, left ventricular ejection fraction; Na, sodium; Cr, creatinine; ACEI, angiotensin converting enzyme inhibitor; ARB, angiotensin II receptor blocker; BB, beta-blocker; AA, aldosterone antagonist.

Table S2. Multivariate Cox proportional-hazards model for the factors associated with 90-day and post-90-day all-cause mortality.

	**Non-ischemic heart failure**				**Ischemic heart failure**			
	**90-day All-cause death**		**Post-90-day All-cause death**		**90-day All-cause death**		**Post-90-day All-cause death**	
	**Adjusted HR (95% CI)**	***p***	**Adjusted HR (95% CI)**	***p***	**Adjusted HR (95% CI)**	***p***	**Adjusted HR (95% CI)**	***p***
**Groups**								
Non-IHF with normal hsTnI	1.00		1.00					
Non-IHF with elevated hsTnI	2.47 (1.81-3.37)	<0.001	1.36 (1.13-1.63)	0.001				
IHF with normal hsTnI					1.00			
IHF with elevated hsTnI					2.91 (1.65-5.12)	<0.001		
**Demographics**								
Age ≥65 years	2.32 (1.75-3.06)	<0.001	2.91 (2.34-3.62)	<0.001	2.35 (1.59-3.48)	<0.001	2.29 (1.73-3.03)	<0.001
Male sex			0.88 (0.74-1.04)	0.142				
**Comorbidities**								
Hypertension							1.24 (0.99-1.55)	0.062
Diabetes								
Previous admission due to HF	1.22 (0.95-1.55)	0.116	1.81 (1.52-2.15)	<0.001			1.33 (1.09-1.61)	0.004
Prior CAD								
Prior MI	2.20 (1.36-3.55)	0.001						
CKD	1.54 (1.13-2.10)	0.006	1.81 (1.44-2.27)	<0.001	0.67 (0.46-0.98)	0.038	1.74 (1.41-2.16)	<0.001
COPD					1.85 (1.31-2.61)	0.001	1.38 (1.03-1.85)	0.032
CVA							1.38 (1.11-1.72)	0.004
Malignancy	1.63 (1.18-2.25)	0.003	1.53 (1.18-1.99)	0.001	1.37 (0.91-2.05)	0.132	1.51 (1.10-2.07)	0.011
**Findings at admission**								
SBP <110 mmHg	2.22 (1.73-2.85)	<0.001			1.56 (1.18-2.07)	0.002		
LVEF <40%					1.69 (1.26-2.27)	0.001	1.46 (1.20-1.79)	<0.001
Serum Na <135 mEq/L	1.66 (1.30-2.12)	<0.001	1.61 (1.33-1.94)	<0.001	1.43 (1.09-1.89)	0.010	1.50 (1.21-1.85)	<0.001
Serum Cr ≥2.0 mg/dL					1.88 (1.33-2.65)	<0.001		
Atrial fibrillation					1.26 (0.92-1.71)	0.148		

**Medication at discharge**						
ACEI/ARB	0.42 (0.33-0.55)	<0.001	0.87 (0.73-1.04)	0.137	0.39 (0.30-0.52)	<0.001
BB	0.54 (0.41-0.70)	<0.001	0.81 (0.69-0.97)	0.019	0.45 (0.34-0.59)	<0.001
AA	0.77 (0.60-0.99)	0.041			0.79 (0.59-1.05)	0.108

HR, hazard ratio; IHF, ischemic heart failure; hsTnI, high-sensitivity troponin-I; HF, heart failure; CKD, chronic kidney disease; COPD, chronic pulmonary obstructive disease; CVA, cerebrovascular accident; CAD, coronary artery disease; MI, myocardial infarction; SBP, systolic blood pressure; LVEF, left ventricular ejection fraction; Na, sodium; Cr, creatinine; ACEI, angiotensin converting enzyme inhibitor; ARB, angiotensin II receptor blocker; BB, beta-blocker; AA, aldosterone antagonist.
